# Improvement of Ethanol Gas-Sensing Responses of ZnO–WO_3_ Composite Nanorods through Annealing Induced Local Phase Transformation

**DOI:** 10.3390/nano9050669

**Published:** 2019-04-30

**Authors:** Yuan-Chang Liang, Che-Wei Chang

**Affiliations:** 1 Institute of Materials Engineering, National Taiwan Ocean University, Keelung 20224, Taiwan; 2 Undergraduate Program in Optoelectronics and Materials Technology, National Taiwan Ocean University, Keelung 20224, Taiwan; jf860218@gmail.com

**Keywords:** sputtering, composite nanorods, phase transformation, annealing

## Abstract

In this study, ZnO–WO_3_ composite nanorods were synthesized through a combination of hydrothermal growth and sputtering method. The structural analysis results revealed that the as-synthesized composite nanorods had a homogeneous coverage of WO_3_ crystallite layer. Moreover, the ZnO–WO_3_ composite nanorods were in a good crystallinity. Further post-annealed the composite nanorods in a hydrogen-containing atmosphere at 400 °C induced the local phase transformation between the ZnO and WO_3_. The ZnO–WO_3_ composite nanorods after annealing engendered the coexistence of ZnWO_4_ and WO_3_ phase in the shell layer which increased the potential barrier number at the interfacial contact region with ZnO. This further enhanced the ethanol gas-sensing response of the pristine ZnO–WO_3_ composite nanorods. The experimental results herein demonstrated a proper thermal annealing procedure of the binary composite nanorods is a promising approach to modulate the gas-sensing behavior the binary oxide composite nanorods.

## 1. Introduction

Composite nanorod systems composed of various binary semiconductor oxides have been shown a promising approach to enhance the gas-sensing properties of the constituent compounds. For example, hydrothermally derived flower-like CeO_2_–SnO_2_ composites exhibit improved trimethylamine gas-sensing response than that of the pristine SnO_2_ [1]. Moreover, hydrogen-sensing properties of ZnO nanofibers are significantly enhanced through NiO loading in a composite structure [2]. SnO_2_/ZnO hetero-nanofibers demonstrate improved acetone gas-sensing responses in comparison with that of the pristine ZnO nanofibers [3]. By tuning the sputtering coated VO_x_ morphology on the one-dimensional ZnO, ZnO–VO_x_ composites demonstrate improved oxidizing gas-sensing responses than that of the pristine ZnO [4].

Among various binary oxides, ZnO is one of the most studied n-type semiconductor oxides which was widely used for gas-sensing material because of its low cost, high chemical stability, and versatile preparation methods. Furthermore, ZnO in a low-dimensional structure is of potential interest for gas sensor device applications because its high specific surface area enables efficient reaction between the oxide surface and target gas molecules [5,6]. However, developing high gas-sensing responses of the ZnO nanostructures toward various target gases is still highly desired and is technically challenging. Various ZnO-based composite systems incorporated with another binary oxide have been proposed to improve the ZnO gas-sensing properties based on the aforementioned demand [2,4,7]. By contrast, WO_3_ is another promising gas-sensing binary oxide. It also has advantages of low cost, high chemical stability, and excellent process-dependent reproducibility. Recent progress has shown that WO_3_ with various morphologies is promising in applications of gas sensors to detect toxic gases [8,9,10]. Moreover, WO_3_ crystals can be synthesized through various physical and chemical methods and the crystalline quality and morphology can be easy controlled through varying the process conditions. [11,12]. Although various ZnO-based composite systems have been proposed to improve the gas-sensing responses to target gases, the reports on construction of one-dimensional ZnO–WO_3_ composite system are still limited in number. Moreover, thermal annealing of solid materials is an efficient method to modulate their microstructures and to control their physical and chemical properties [13,14]. The past research works show that conducting proper thermal annealing procedures causes the possible solid-state reaction between the constituent oxides in a nanoscale in low-dimensional oxide systems [15,16]. The existence of phase transformation between the binary oxides in a low-dimensional composite system modifies the electric properties of the original composite systems without thermal annealing procedures. This broadens the design of the functionality and changes property performance of the pristine oxide composite system.

## 2. Materials and Methods

In this study, ZnO-based composite nanorods coated with the WO_3_ and ZnWO_4_ shell layers were synthesized through a combinational methodology of hydrothermal growth and sputtering. Hydrothermally synthesized high-density ZnO nanorods were used as templates for growing the ZnO-based composite nanorods. The hydrothermal growth reactions of the ZnO nanorods were conducted at 95 °C for 9 h. The detailed process procedures were reported elsewhere [14]. During sputtering growth of the WO_3_ shell layer, the sputtering power of tungsten metallic target was fixed at 80 W. The thin-film growth temperature was maintained at 375 °C with an Ar/O_2_ ratio of 3:2. Then, the as-synthesized ZnO–WO_3_ composite nanorods were subsequently annealed in a 95% N_2_/5% H_2_ atmosphere for 20 min at the temperatures of 400~500 °C to induce a solid-state reaction between the ZnO surface and WO_3_ ultra-thin layer.

The scanning electron microscopy (SEM; Hitachi S-4800, Tokyo, Japan) was used to investigate the surface morphology of nanorod samples. X-ray diffraction (XRD; Bruker D2 PHASER, Karlsruhe, Germany) was further used to investigate crystallographic structures of the samples. Moreover, the detailed microstructures of the composite nanorods with and without the thermal annealing were characterized by high-resolution transmission electron microscopy (HRTEM; Philips Tecnai F20 G2, Amsterdam, The Netherland). X-ray photoelectron spectroscope (XPS; PHI 5000 VersaProbe, Chigasaki, Japan) analysis was performed to determine the chemical binding states of the constituent elements of the composite nanorods. Silver contact electrodes were formed on the surface of the nanorod samples for gas-sensing measurements. The ethanol vapor concentrations of 25–500 ppm were used as target gas. The dry air was used as carrier gas herein. The variation of sensor resistance before and after introducing ethanol vapor was recorded. The gas-sensing response of the sensors to ethanol vapor herein is defined as the R_a_/R_g_, which is the electric resistance ratio of the resistance of the gas sensor in the absence of ethanol vapor to the resistance of the sensor in ethanol vapor.

## 3. Results

**Figure 1:**
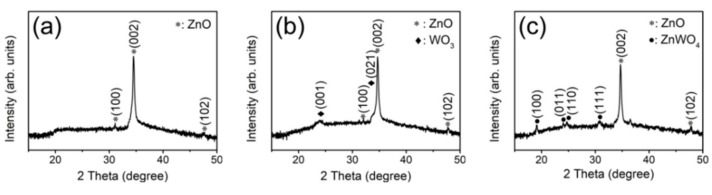
X-ray diffraction (XRD) patterns: (**a**) ZnO nanorods, (**b**) ZnO–WO_3_ composite nanorods, (**c**) ZnO–WO_3_ composite nanorods annealed at 400 °C.

The crystallographic structures of the as-prepared ZnO nanorods, ZnO–WO_3_ nanorods with and without thermal annealing at 400 °C were identified using XRD measurements (Figure 1). Figure 1a shows the XRD pattern of the ZnO nanorods which were used as a template for preparing various composite nanorods. The Bragg reflections in Figure 1a demonstrate that the ZnO nanorods has a hexagonal wurtzite structure and exhibits preferred (002) orientation (JCPDS no. 005-0664). The XRD pattern of the ZnO nanorods in situ sputtering coated with WO_3_ thin films was exhibited in Figure 1b. The Bragg reflections centered approximately 24.5° and 34.2° are ascribed to (001) and (201) crystallographic planes of orthorhombic WO_3_ (JCPDS no. 20-1324), respectively. Well crystalline ZnO–WO_3_ composite nanorods were successfully formed via sputtering WO_3_ thin films onto the surfaces of the ZnO nanorods. Figure 1c shows the XRD patterns of the ZnO–WO_3_ nanorods annealed at 400 °C. Figure 1c shows that the WO_3_ peaks are disappeared completely after the annealing procedure and several new diffraction peaks are observed at approximately 19.6°, 24.5°, 25.3° and 31.3° which are assigned to (100), (011), (110) and (111) of monoclinic ZnWO_4_ (JCPDS no. 15-0774). The XRD result transformation of ultra-thin WO_3_ thin film with the ZnO into the ternary ZnWO_4_ phase occurred after the thermal annealing procedure. M. Bonanni et al. reported that the solid-state reaction between ZnO and WO_3_ starts to develop at 350 °C [17]. The annealing temperature of 400 °C herein might have a sufficient thermal energy to activate the phase transformation between the WO_3_ and ZnO of the ZnO–WO_3_ composite nanorods. The XRD results revealed the ZnWO_4_ layers with a polycrystalline feature were formed on the surfaces of residual ZnO nanorods after the annealing procedure.

**Figure 2:**
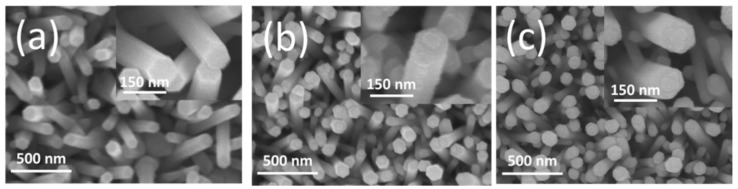
Scanning electron microscopy (SEM) micrographs: (**a**) ZnO nanorods, (**b**) ZnO–WO_3_ composite nanorods, (**c**) ZnO–WO_3_ composite nanorods annealed at 400 °C.

Figure 2 shows SEM images of the ZnO, ZnO–WO_3_ nanorods with and without annealing at 400 °C. Figure 2a shows a typical SEM image of as-synthesized ZnO nanorods, revealing a hexagonal crystal featured cross-section of the ZnO nanorods with a diameter in the range of 85–100 nm. The surface of the ZnO nanorods was smooth. Figure 2b presents the SEM image of ZnO nanorods after sputtering coated with ultra-thin WO_3_ thin film. Compared to the bare ZnO nanorods, the surface feature of the ZnO–WO_3_ composite nanorods became more rugged when the WO_3_ crystallites were decorated onto the surfaces of the ZnO nanorods. Notably, the hexagonal cross-sectioned morphology of the ZnO nanorods was maintained after coating the WO_3_ thin film, revealing the deposition of the ultra-thin WO_3_ layer on the ZnO nanorods’ surfaces. Figure 2c shows the ZnO–WO_3_ composite nanorods annealed at 400 °C. The ZnO–WO_3_ composite nanorods annealed at 400 °C did not exhibit substantial morphology change. The composite nanorods maintained a visible hexagonal cross-sectional crystal feature. By contrast, a high solid-state reaction temperature above 600 °C involves the marked surface roughening process of oxide composite nanorods; this was observed in phase transformation of other oxide nanocomposite systems [16].

**Figure 3:**
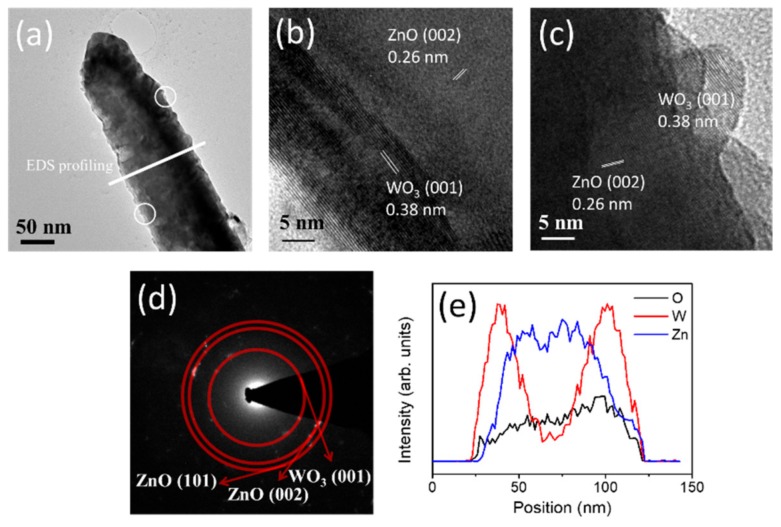
Transmission electron microscopy (TEM) analysis of the ZnO–WO_3_ composite nanorod: (**a**) low-magnification image, (**b**,**c**) high-resolution images from local regions, (**d**) selected area electron diffraction (SAED) pattern, (**e**) line-scan profiling spectra across the composite nanorod.

Figure 3a shows a low-magnification TEM image of a ZnO nanorod coated with a thin WO_3_ layer. The surface of the composite nanorod exhibited an uneven feature. Figure 3b,c demonstrate high-resolution TEM (HRTEM) images of a ZnO–WO_3_ composite nanorod taken from the different positions at the WO_3_/ZnO interface. Figure 3b reveals an ultra-thin WO_3_ layer covered on the surface of the nanorod and the interface between the ZnO nanorod and WO_3_ layer is abrupt. By contrast in Figure 3c, tiny, nanoscaled surface bumps appeared on the surface of the composite nanorod. This might engender the uneven surface feature of the composite nanorod. The sputtering growth of binary oxides at an elevated temperature is likely to form island- or bump-like crystals on the hetero-substrates [4,7]. The in situ sputtering growth of WO_3_ crystals onto the surfaces of the ZnO nanorods at 375 °C herein might cause locally inhomogeneous crystal growth and formed WO_3_ bumps on the ZnO nanorods. The ordered lattice fringes in the outer region of the composite nanorod revealed the coverage of well-crystallized WO_3_ crystals on the surface of the nanorod. The lattice fringe spacing of approximately 0.38 nm corresponds to the interplanar distance of orthorhombic WO_3_ (001). Furthermore, the lattice fringe spacing of approximately 0.26 nm in the figures demonstrated the interplanar distance of hexagonal ZnO (002). The selected area electron diffraction (SAED) pattern taken from several ZnO–WO_3_ composite nanorods revealed the crystalline feature and a composite structure of the hexagonal ZnO nanorods sputtering coated with the orthorhombic WO_3_ thin film. Furthermore, elemental line-scan profiles of Zn, W, and O elements across the ZnO–WO_3_ composite nanorod were displayed in Figure 3e. The line-scan profiles revealed that the W element was well distributed on the surface of the ZnO nanorod, revealing a formation of the compositionally defined composite structure of core-ZnO and shell-WO_3_.

**Figure 4:**
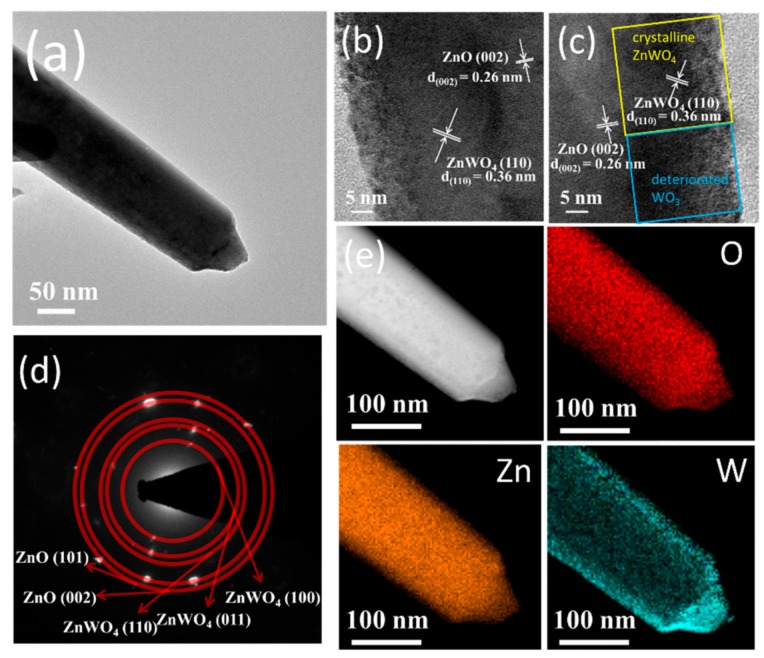
TEM analysis of the ZnO–WO_3_ composite nanorod annealed at 400 °C: (**a**) low-magnification image, (**b**,**c**) high-resolution images from local regions, (**d**) SAED pattern, (**e**) elemental mapping images of the nanorod.

Figure 4a shows a low-magnification TEM image of a ZnO–WO_3_ composite nanorod with a thermal annealing at 400 °C. HRTEM images of the composite taken from various interfacial regions are demonstrated in Figure 4b,c. In Figure 4b, well-ordered and long-range arrangement of lattice fringes appear at the outer region of the composite nanorod. Moreover, the ordered lattice fringes arranged in the other orientation were found in the inner region of the composite nanorod. The lattice fringes with a spacing of approximately 0.36 nm in the outer region of the composite nanorod were attributed to the interplanar distance of monoclinic ZnWO_4_ (110). By contrast, the lattice fringes with a spacing of 0.26 nm in the inner region of the composite nanorod were assigned to the interplanar distance of hexagonal ZnO (002). Figure 4b reveals that the WO_3_ phase in the outer region of the ZnO–WO_3_ composite nanorod transforms into a ternary phase of ZnWO_4_ through a solid-state reaction process during the post-annealing procedure in this study. The ZnWO_4_ crystals exhibited a good crystalline feature on the outer region of the composite nanorod and the interface of the ZnWO_4_ and ZnO phase was sharp. However, the mixed lattice fringes arrangements were observed in the outer region of the composite nanorod in Figure 4c. In addition to the ordered lattice fringes which originated from the ZnWO4 (110) as indexed in the figure, some local region demonstrated that the lattice fringes were arranged in a slightly chaotic state. This revealed the presence of crystalline ZnWO_4_ and deteriorated WO_3_ crystals in the outer region of the composite nanorod. The observation of the TEM analysis demonstrated that most WO_3_ crystals transformed into crystalline ZnWO_4_ after annealing; whereas, partial WO_3_ crystals did not yield the phase transformation with the ZnO due to the insufficient reaction condition. Moreover, the residual WO_3_ phase region demonstrated the deteriorated crystallinity after annealing because of the presence of hydrogen in the annealing atmosphere. In conclusion, the composite nanorod is mainly composed of crystalline ZnWO_4_ phase with a larger range and spatially distributed residual WO_3_ phase in a smaller content in the outer layer. The SAED pattern taken from the several composite nanorods in Figure 4d indicate the various groups of diffraction rings, suggesting the presence of crystalline ZnO and ZnWO_4_ in the composite nanorods. The elemental mapping images were further used to analyze the distribution of Zn, W, and O in a single ZnO–WO_3_ composite nanorod treated with a thermal annealing at 400 °C (Figure 4e). The EDS mapping images clearly identified the spatial distributions of Zn, W, and O in the composite structure. The Zn and O elements existed the entire area of the nanorod. In particular, W is the main element distributed in the outer shell of the composite nanorod. TEM results indicated that a solid-state reaction occurred at the interface of WO_3_/ZnO and formed a new shell layer phase of ZnWO_4_ on the surface of the residual ZnO core in the composite nanorod. Similar solid-state reaction between the different binary oxides in low-dimensional systems to form a ternary phase in the outer region of the composite nanorods has also been reported in ZnO–SnO_2_ and ZnO–TiO_2_ [16,18].

**Figure 5:**
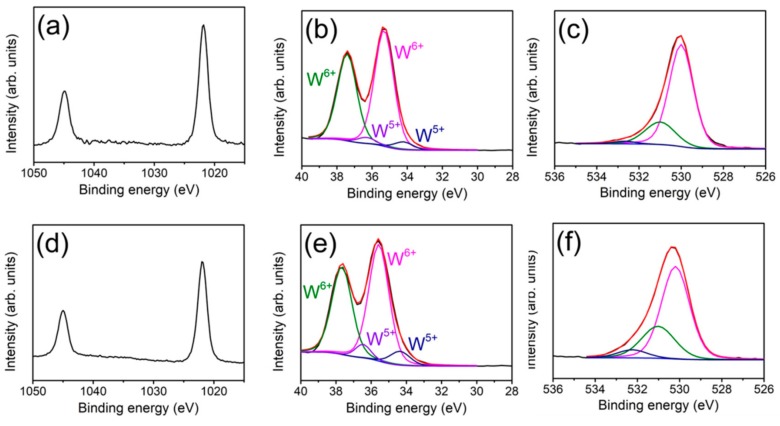
X-ray photoelectron spectroscope (XPS) analysis of the ZnO–WO_3_ composite nanorods: (**a**) Zn 2p, (**b**) W 4f, (**c**) O1s. XPS analysis of the ZnO–WO_3_ composite nanorods after annealing: (**d**) Zn 2p, (**e**) W 4f, (**f**) O1s.

The elemental binding states of the ZnO–WO_3_ composite with and without a thermal annealing at 400 °C were investigated by XPS. The narrow spectra of Zn 2p, W 4f, and O 1s for the ZnO–WO_3_ composite nanorods are recorded in Figure 5a–c. Zn 2p spectrum of the composite nanorods in Figure 5a shows two peaks at approximately 1044.8 eV and 1021.8 eV which are respectively attributed to Zn 2p_1/2_ and Zn 2p_3/2_ and suggest the presence of Zn^2+^ ions in the oxide [16]. Moreover, the W4 f spectrum (Figure 5b) of pristine ZnO–WO_3_ nanorods consisted of two spin-orbit doublets corresponding to the different valence states of tungsten. The bigger doublet located at 37.4 eV and 35.3 eV is assigned to W 4f_5/2_ and W 4f_7/2_ of W^6+^, respectively and the smaller one is allocated to W^5+^ [11]. No metallic W component was detected from the sample. The asymmetric O 1s spectrum of ZnO–WO_3_ composite nanorods was displayed in Figure 5c and that spectrum was deconvoluted into three subpeaks at approximately 530.1 eV, 530.9 eV, and 531.9 eV, matching the oxygen coordination in lattice oxygen, vacancy oxygen, and surface chemisorbed oxygen, respectively [18,19]. By contrast, in Figure 5d, the Zn 2p spectrum of the ZnO–WO_3_ composite nanorods with a thermal annealing at 400 °C exhibited a similar spectrum feature as exhibited in Figure 5a, revealing the divalent state of the zinc in the nanorods. Figure 5e shows the W4f spectrum of the composite nanorods annealed in a hydrogen-contained atmosphere. Notably, even annealed in oxygen deficient atmosphere, the metallic W component was not detected on the surfaces of the composite nanorods under the given annealing condition. Comparatively, the W 4f spectrum with the deconvoluted peaks in Figure 5e exhibited that the ZnO–WO_3_ composite nanorods with the thermal annealing procedure demonstrated the area ratio of W^5+^ spin-orbit doublet becomes larger, resulting from the existence of the crystal deterioration region in the shell oxide layer. The O1s spectrum of the corresponding sample was shown in Figure 5f and was further used to explain the W4f XPS result. The O 1s spectrum from the composite nanorods with a thermal annealing process showed a marked intensity decrease in the lattice oxygen subpeak and a relative intensity rise in the subpeaks associated with oxygen vacancy and chemisorbed oxygen, compared with those from the ZnO–WO_3_ composite nanorods without a thermal annealing. The concentration of oxygen vacancies has a direct relationship with the state of the oxide’s crystallinity. A similar phenomenon of increased oxygen vacancies in oxides annealed in hydrogen-contained atmosphere has been proposed in previous works [20]. A higher degree of oxygen deficiency in the outer region of the composite nanorods engendered a larger content of W^5+^ in the tungsten-based oxides of the composite nanorods. The XPS results herein demonstrated that the annealing temperature of 400 °C for the ZnO–WO_3_ composite nanorods engendered more oxygen vacancies in the surfaces; the tungsten was still in an oxide binding status without reducing to the metallic binding form.

**Figure 6:**
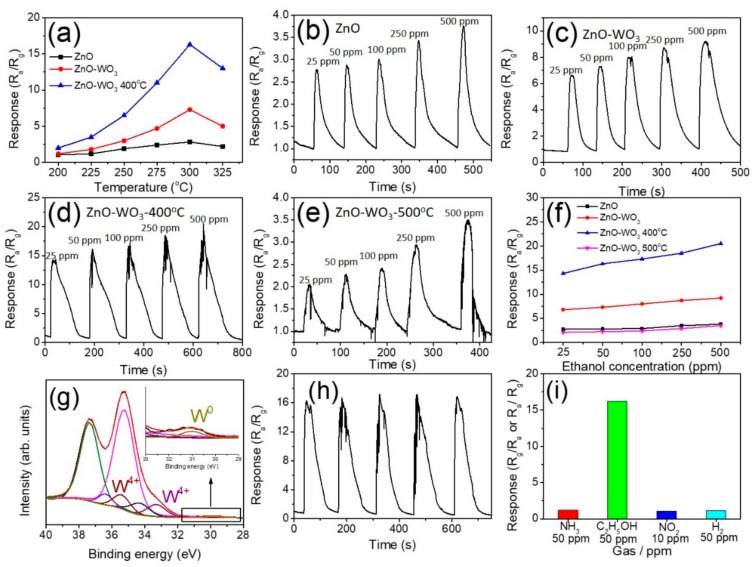
(**a**) Gas-sensing response vs. temperature curves of various nanorod sensors on exposure to 50 ppm ethanol vapor. Dynamic gas-sensing response curves of sensor on exposure to 25–500 ppm ethanol vapor: (**b**) ZnO nanorods, (**c**) ZnO–WO_3_ composite nanorods, (**d**), ZnO–WO_3_ composite nanorods annealed at 400 °C, (**e**) ZnO–WO_3_ composite nanorods annealed 500 °C. (**f**) Gas-sensing response vs. ethanol vapor concentration curves. (**g**) XPS W 4f spectrum of the composite nanorods annealed at 500 °C. (**h**) Cycling testes of the composite nanorods annealed at 400 °C exposed to 50 ppm ethanol vapor. (**i**) Selectivity tests of the composite nanorods annealed at 400 °C.

The optimal operating temperature with the highest gas-sensing response for the various nanorods was determined. The gas-sensing responses of all samples on exposure to 50 ppm ethanol vapor were measured at the temperature range of 200–325 °C (Figure 6a). For the ZnO nanorods, the gas-sensing response to 50 ppm ethanol vapor varied from 1.1 to 2.8 corresponding with the temperature from 200 to 325 °C (black curve in Figure 6a). The gas-sensing responses of ZnO–WO_3_ composite nanorods were from 1.3 to 7.3 (red curve in Figure 6a), which was substantially higher than that of the ZnO nanorods at all tested temperatures. Moreover, the gas-sensing responses of ZnO–WO_3_ composite nanorods annealed at 400 °C ranged from 2.1 to 16.2 (blue curve in Figure 6a), which demonstrated the highest response among the samples at the tested temperatures. Significantly, the nanorod sensors herein exhibited the maximum gas-sensing responses to ethanol at 300 °C, suggesting that a resultant equilibrium between surface reaction with ethanol vapor molecules and the diffusion of ethanol vapor molecules to the nanorods’ surfaces occurred at 300 °C [9]. Figure 6b–d show the dynamic response curves of ZnO nanorods, ZnO–WO_3_ nanorods, and ZnO–WO_3_ nanorods annealed at 400 °C, respectively, exposed to 25–500 ppm ethanol vapor at the operating temperature of 300 °C. For comparison, gas-sensing tests of the ZnO–WO_3_ composite nanorods annealed at 500 °C were also conducted to evaluate whether the higher annealing temperature improves the gas-sensing performance of the initially-synthesized ZnO–WO_3_ composite nanorods (Figure 6e). All nanorod samples exhibited reversible and stable response and recovery behaviors during gas-sensing tests. The nanorod samples herein showed a typical n-type sensing behavior because of the n-type conduction nature of the constituent oxides. The gas-sensing responses of the sensors made from various nanorods on exposure to various ethanol concentrations were summarized in Figure 6f. The WO_3_-decorated ZnO nanorod sensor exhibited much higher responses than the pristine ZnO. Furthermore, the ZnO–WO_3_ composite nanorods annealed at 400 °C showed the highest response in all ethanol vapor concentrations. Notably, the sensing ability of the ZnO–WO_3_ composite nanorods annealed at the higher temperature of 500 °C was substantially weakened. To confirm the possible reason for the deterioration of the gas-sensing ability, XPS measurements were conducted. Figure 6g demonstrates that the W4f spectrum include not only W^6+^ and W^5+^ but also W^4+^ and W^0^ in the ZnO–WO_3_ composite nanorods annealed at 500 °C. The subpeaks located at 33.4 eV and 35.5 eV are ascribed to tetravalent bond of tungsten and that at 31.1 eV was associated with the contribution of metallic tungsten [21,22]. The existence of mixed binding states of tungsten implied that substantial deoxidization of the WO_3_ shell layer occurred during the high-temperature annealing in the hydrogen-contained atmosphere. The appearance of metallic W component revealed that the WO_3_ is not in a pure oxide phase and this might deteriorate the gas-sensing performance of the n-type WO_3_ oxide. A similar deoxidization of metal oxides annealed in the hydrogen-contained atmosphere at a high temperature has been proposed and resultant deteriorated electric properties of the metal oxides are involved [20,23]. The reproducibility and stability of the sensor made from the ZnO–WO_3_ nanorods annealed at 400 °C were further examined at its optimum operating temperature of 300 °C to 50 ppm ethanol vapor concentration (Figure 6h). The difference obtained in the gas response values of the sensor after cycling tests was very small and hence negligible. This suggests that the proposed composite nanorod sensor showed good reproducibility to detect ethanol vapor. Figure 6i shows the selectivity of the gas sensor based on the ZnO–WO_3_ nanorods annealed at 400 °C. The sensor was exposed to ammonia gas, ethanol vapor, nitrogen dioxide gas, and hydrogen gas of the appropriate concentrations at 300 °C, respectively. It can be seen that the sensor exhibited the substantially highest response to ethanol vapor, revealing its suitability for detecting ethanol vapor in the test environment. Table 1 compares the gas-sensing responses of various ZnO-based composites exposed to appropriate ethanol vapor concentrations at 300 °C [24,25,26]. The ZnO–WO_3_ composite nanorods annealed at 400 °C in this study presented superior ethanol vapor detecting ability among the various reference works.

## 4. Discussion

The possible reasons caused various gas-sensing responses of the ZnO nanorods and various composite nanorods were further explained with the space-charge layer model [1,27]. In ambient air, oxygen molecules were absorbed on surfaces of the composite nanorods. These oxygen molecules become surface absorbed oxygen species (such as O^−^_(ads)_ and O^2−^_(ads)_) by capturing free electrons from the conducting bands of the oxides at the elevated sensor operating temperature of 300 °C. The reactions are described as follows:

O_2_ (ambient) → O_2(ads)_ (n-type oxides) (1)

O_2 (ads)_ + 2e^−^ → 2O^−^_(ads)_ (2)

O^−^_(ads)_ + e^−^ → O^2−^_(ads)_ (3)

In this process, an electron depletion layer will be formed on the surfaces of the composite nanorods, resulting in a decrease of carrier concentration and an increase of sensor resistance. When the oxide nanorod sensor was exposed to ethanol vapor, the absorbed oxygen species will react with ethanol molecules according to the following possible reactions:

C_2_H_5_OH + 6O^−^ → 2CO_2_ + 3H_2_O + 6e^−^ (4)

C_2_H_5_OH + 6O^2−^ → 2CO_2_ + 3H_2_O + 12e^−^ (5)

**Figure 7:**
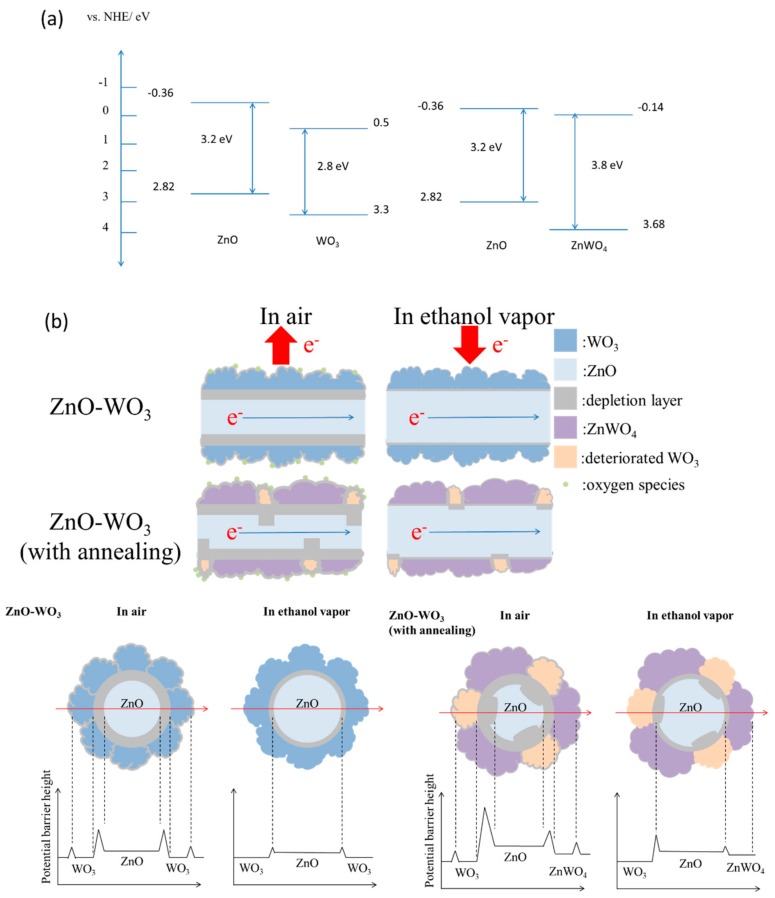
(**a**) Energy band alignments of ZnO/WO_3_ and ZnO/ZnWO_4_. (**b**) Possible ethanol gas-sensing mechanisms of the ZnO–WO_3_ composite nanorods with and without a thermal annealing at 400 °C. The corresponding cross-sectional views for the possible potential barrier height variation was also shown in the bottom region of the plot.

As a result, the electrons trapped in the oxygen species are released back into the conduction band, leading to a decrease of the thickness of the depletion layer and the resistance of the oxides. In addition to the surface depletion layer, the contact of the different oxides in a one-dimensional heterostructure system engenders formation of interfacial depletion regions because of different work functions of the adjacent oxides. A proposed energy band structure diagram of the ZnO/WO_3_ and ZnO/ZnWO_4_ heterojunction herein are shown in Figure 7a [28,29]. The electrons will flow from ZnO nanorod to outer WO_3_ (or ZnWO_4_) crystals in the ZnO/WO_3_ (or ZnO/ZnWO_4_) heterostructures until their Fermi levels are equalized. Therefore, the exposure of the composite nanorods in air ambient result in formation of surface depletion regions in the surface WO_3_ (or ZnWO_4_) crystals and interfacial depletion regions inside the ZnO nanorod for the proposed various composite nanorods. This process creates an electron depletion layer on the surface of the ZnO core material and further bended the energy band and lead to a higher resistance of the composite nanorods with and without thermal annealing. The formation of additional interfacial depletion regions increased the potential barrier number in the composite nanorods than in the pristine ZnO nanorods; therefore, a larger resistance variation degree of the composite nanorods than that of the ZnO nanorods on exposure to the ethanol vapor is observed. This explained that the higher ethanol vapor sensing responses of the composite nanorods than those of the ZnO nanorods under the given gas-sensing tests herein. Similarly, a heterogeneous structure that improved the gas-sensing behavior of one-dimensional n-type oxide nanorods was demonstrated in ZnO–SnO_2_, ZnO–Zn_2_SnO_4_, and TiO_2_–CdO on exposure to test gases [16,19]. Comparatively, the ZnO–WO_3_ nanorods with a thermal annealing at 400 °C substantially enhanced their ethanol gas-sensing responses at the given test conditions. This improvement of the gas-sensing response of the composite nanorods annealed at 400 °C might be attributed to the random thickness of the depletion layers of the surface and interface regions of the composite nanorods resulting from the local phase transformation of the ZnO–WO_3_ composite system during the annealing process herein (Figure 7b). Compared to the chemically homogeneous shell layer of ZnO–WO_3_ nanorods, the ZnO–WO_3_ nanorods with a thermal annealing at 400 °C had a composite shell layer structure consisted of crystalline ZnWO_4_ and deteriorated WO_3_ as revealed in the structural analysis results. It is expected that the potential barrier number of the ZnO–WO_3_ composite nanorods annealed at 400 °C was higher than that of the composite nanorods without a thermal annealing. Three types of depletion regions included surface depletions in the deteriorated WO_3_ and crystalline ZnWO_4_, depletion regions at WO_3_/ZnWO_4_ boundaries of the shell layer and interfacial depletion regions at the ZnO/WO_3_ and ZnO/ZnWO_4_ are expected to exist in the ZnO–WO_3_ composite nanorods annealed at 400 °C. The local phase transformation of the ZnO/WO_3_ after thermal annealing at 400 °C for the ZnO–WO_3_ composite nanorod system in this study created a higher number of the potential barriers in the composite system as exhibited in Figure 7b. An increased potential barrier number in the composite systems has shown a substantial drop degree of the sensor resistance on exposure to the reducing gases and therefore this resulted in an enhanced gas-sensing response [3,9]. The cross-sectional potential barrier height variation alone the guided arrow red line in Figure 7b demonstrated that a more complex potential barrier height variation before and after introducing the ethanol vapor will be expected for the ZnO–WO_3_ composite nanorods with an annealing procedure. Comparatively, further introducing the reducing vapor of ethanol into the test chamber, the injection of electrons from the adsorbed oxygen ions into the conduction bands of the constituent oxides of the composite nanorods caused a larger degree of resistance variation of the composite nanorods with thermal annealing because of their diverse microstructures in the composite system. The substantial microstructural differences in the ZnO–WO_3_ composite nanorods with and without thermal annealing at 400 °C herein supported the different ethanol vapor sensing responses of various composite nanorods.

## 5. Conclusions

A combinational methodology of hydrothermal growth and sputtering was used to synthesize ZnO–WO_3_ composite nanorods. Furthermore, a thermal annealing procedure was conducted in a hydrogen-contained atmosphere to induce a microstructural modification of the composite nanorods. The structural analysis revealed that the ZnO nanorods sputtering coated with the ultra-thin WO_3_ thin film formed well crystalline ZnO–WO_3_ composite nanorods. The thermal annealing procedure at 400 °C further engendered the formation of ternary ZnWO_4_ phase and deteriorated WO_3_ phase on the surfaces of the ZnO nanorods. The ethanol gas-sensing test results demonstrated that the construction of the ZnO–WO_3_ composite nanorods is advantageous for improving the gas-sensing response of the ZnO nanorods to ethanol vapor. The formation of the heterogeneous junction between the ZnO and WO_3_ contributed to the enhanced ethanol gas-sensing responses. Moreover, an increase of potential barrier number in the ZnO–WO_3_ composite nanorods annealed at 400 °C improved the gas-sensing responses of the composite nanorods without a thermal annealing. The composite nanorods annealed at 400 °C exhibited a strong response of 16.2 at the gas concentration of 50 ppm, while the pristine ZnO–WO_3_ could only reach 7.3 at the identical gas concentration. Such intriguing ethanol gas-sensing response enhancement could be ascribed to the existence of heterogeneous junctions at interfaces of ZnO/ZnWO_4_, ZnO/WO_3_, and ZnWO_4_/WO_3_ in the composite nanorods after annealing at 400 °C. The local structural modification of the composite nanorods through a proper thermal annealing condition is feasible to control the gas-sensing behavior of the oxide composite nanorods. Moreover, the ZnO–WO_3_ nanorods annealed at 400 °C exhibited high selectivity to ethanol vapor among the various target gases of NH_3_, H_2_, and NO_2_. This composite nanorod system is of potential to effectively detect ethanol vapor in an open environment.

## Figures and Tables

**Table 1:** Comparison of ethanol gas-sensing responses of various ZnO-based heterogeneous sensors at 300 °C [24,25,26].

Materials	Preparation Method	Operating Temperature (°C)	Concentration (ppm)	Response
ZnO/NiO	Spray pyrolysis & Chemical bath	300	500	1.5
ZnO/SnO_2_	Hydrothermal	300	50	11.2
ZnO/g-C_3_N_4_	Solvothermal treatment	300	100	9.2
ZnO/WO_3_ annealed (this work)	Hydrothermal & sputtering	300	50	16.2

## References

[1] Xue D., Wang Y., Cao J., Zhang Z. (2018). Hydrothermal Synthesis of CeO_2_-SnO_2_ Nanoflowers for Improving Triethylamine Gas Sensing Property. Nanomaterials.

[2] Lee J.-H., Kim J.-Y., Mirzaei A., Kim H.W., Kim S.S. (2018). Significant Enhancement of Hydrogen-Sensing Properties of ZnO Nanofibers through NiO Loading. Nanomaterials.

[3] Du H., Li X., Yao P., Wang J., Sun Y., Dong L. (2018). Zinc Oxide Coated Tin Oxide Nanofibers for Improved Selective Acetone Sensing. Nanomaterials.

[4] Liang Y.C., Cheng Y.R. (2015). Combinational physical synthesis methodology and crystal features correlated with oxidizing gas detection ability of one-dimensional ZnO–VO*_x_* crystalline hybrids. CrystEngComm.

[5] Wang P.P., Qi Q., Zou X., Zhao J., Xuan R.F., Li G.D. (2013). A precursor route to porous ZnO nanotubes with superior gas sensing properties. RSC Adv..

[6] Baratto C. (2018). Growth and properties of ZnO nanorods by RF sputtering for detection of toxic gases. RSC Adv..

[7] Liang Y.C., Lin T.Y., Lee C.M. (2015). Crystal growth and shell layer crystal-feature-dependent sensing and photoactivity performance of zinc oxide-indium oxide core-sehll nanorod heterostructures. CrystEngComm.

[8] Xue D., Wang J., Wang Y., Sun G., Cao J., Bala H., Zhang Z. (2019). Enhanced Methane Sensing Properties of WO_3_ Nanosheets with Dominant Exposed (200) Facet via Loading of SnO_2_ Nanoparticles. Nanomaterials.

[9] Liang Y.C., Chao Y. (2019). Crystal phase content-dependent functionality of dual phase SnO_2_–WO_3_ nanocomposite films via cosputtering crystal growth. RSC Adv..

[10] Fang W., Yang Y., Yu H., Dong X., Wang T., Wang J., Liu Z., Zhao B., Yang M. (2016). One-step synthesis of flower-shaped WO_3_ nanostructures for a high-sensitivity room-temperature NO_x_ gas sensor. RSC Adv..

[11] Liang Y.-C., Chang C.-W. (2019). Preparation of Orthorhombic WO_3_ Thin Films and Their Crystal Quality-Dependent Dye Photodegradation Ability. Coatings.

[12] Wang J.M., Sun X.W., Jiao Z. (2010). Application of Nanostructures in Electrochromic Materials and Devices: Recent Progress. Materials.

[13] Liang Y.C., Zhong H. (2013). Materials synthesis and annealing-induced changes of microstructure and physical properties of one-dimensional perovskite-wurtzite oxide heterostructures. Appl. Surf. Sci..

[14] Liang Y.C., Liao W.K., Liu S.L. (2014). Performance enhancement of humidity sensors made from oxide heterostructure nanorods via microstructural modifications. RSC Adv..

[15] Liang Y.C., Liao W.K. (2014). Annealing induced solid-state structure dependent performance of ultraviolet photodetectors made from binary oxide-based nanocomposites. RSC Adv..

[16] Liang Y.C., Lo Y.J. (2017). High-temperature solid-state reaction induced structure modifications and associated photoactivity and gas-sensing performance of binary oxide one-dimensional composite system. RSC Adv..

[17] Bonanni M., Spanhel L., Lerch M., Fuglein E., Muller G. (1998). Conversion of Colloidal ZnO−WO_3_ Heteroaggregates into Strongly Blue Luminescing ZnWO_4_ Xerogels and Films. Chem. Mater..

[18] Liang Y.C., Hua C.Y., Liang Y.C. (2012). Crystallographic phase evolution of ternary Zn-Ti-O nanomaterials during high-temperature annealing of ZnO-TiO_2_ nanocomposites. CrystEngComm.

[19] Liang Y.C., Xu N.C., Wang C.C., Wei D.W. (2017). Fabrication of Nanosized Island-Like CdO Crystallites-Decorated TiO_2_ Rod Nanocomposites via a Combinational Methodology and Their Low-Concentration NO_2_ Gas-Sensing Behavior. Materials.

[20] Liang Y.C. (2010). Hydrogen-induced degradation in physical properties of dielectric-enhanced Ba_0.6_Sr_0.4_TiO_3_/SrTiO_3_ artificial superlattices. Electrochem. Solid State Lett..

[21] Hussain T., Al-Kuhaili M.F., Durrani S.M.A., Qurashi A., Qayyum H.A. (2017). Enhancement in the solar light harvesting ability of tungsten oxide thin films by annealing in vacuum and hydrogen. Int. J. Hydrogen Energy.

[22] Xie F.Y., Gong L., Liu X., Tao Y.T., Zhang W.H., Chen S.H., Meng H., Chen J. (2012). XPS studies on surface reduction of tungsten oxide nanowire film by Ar^+^ bombardment. J Electron. Spectros. Relat. Phenomena.

[23] Liang Y.C. (2011). Forming gas annealing induced degradation in nanoscale electrical homogeneity of bismuth ferrite thin films. J. Electrochem. Soc..

[24] Li D., Zhang Y., Liu D., Yao S., Liu F., Wang B., Sun P., Gao Y., Chuai X., Lu G. (2016). Hierarchical core/shell ZnO/NiO nanoheterojunctions synthesized by ultrasonic spray pyrolysis and their gas-sensing performance. CrystEngComm.

[25] Yang X., Zhang S., Yu Q., Zhao L., Sun P., Wang T., Liu F., Yan X., Gao Y., Liang X. (2019). One step synthesis of branched SnO_2_/ZnO heterostructures and their enhanced gas-sensing properties. Sens. Actuator B Chem..

[26] Wang L., Liu H., Fu H., Wang Y. (2018). Polymer g-C_3_N_4_ wrapping bundle-like ZnO nanorod heterostructures with enhanced gas sensing properties. J. Mater. Res..

[27] Liang Y.C., Lee C.M., Lo Y.J. (2017). Reducing gas-sensing performance of Ce-doped SnO_2_ thin films through a cosputtering method. RSC Adv..

[28] Jiang X., Zhao X., Duan L., Shen H., Liu H., Hou T., Wang F. (2016). Enhanced photoluminescence and photocatalytic activity of ZnO-ZnWO_4_ nanocomposites synthesized by a precipitation method. Ceram Int..

[29] Lam S.-M., Sin J.-C., Abdullah A.Z., Mohamed A.R. (2014). Transition metal oxide loaded ZnO nanorods: Preparation, characterization and their UV–vis photocatalytic activities. Sep. Purif. Technol..

